# (3-Amino­phen­yl)diphenyl­phosphine oxide–2-propanol (1/1)

**DOI:** 10.1107/S1600536808004674

**Published:** 2008-02-22

**Authors:** Hossein Mahdavi, Javad Amani, Jafar Attar Gharamaleki

**Affiliations:** a School of Chemistry, University College of Science, University of Tehran, PO Box 14155-645, Tehran, Iran; b Faculty of Chemistry, Tarbiat Moallem University, Tehran, Iran

## Abstract

The title compound, C_18_H_16_NOP·C_3_H_8_O, was synthesized by the reduction of (3-nitro­phen­yl)diphenyl­phosphine oxide in the presence of 2-propanol as recrystallization solvent. There are two molecules in the asymmetric unit. Each P atom is tetra­coordinated by three C and one O atom from two phenyl fragments, one aniline group and one double-bonded O atom in a distorted tetra­hedral geometry. C—H⋯π and N—H⋯π inter­actions are present. In the crystal structure, a wide range of non-covalent inter­actions consisting of hydrogen bonding [of the types of O—H⋯O, N—H⋯O and C—H⋯O, with *D*⋯*A* distances ranging from 2.680 (3) to 3.478 (3) Å] and π–π [centroid–centroid distance of 3.7720 (15) Å] stacking inter­actions connect the various components into a supra­molecular structure.

## Related literature

For related literature, see: Aghabozorg *et al.* (2007 ▶); Aghabozorg *et al.* (2008 ▶); Al-Farhan (1992 ▶); Chael & Buchmeiser (2003 ▶); Mahdavi & Amani (2008 ▶).
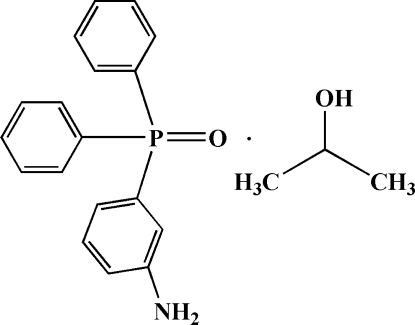


## Experimental

### 

#### Crystal data

C_18_H_16_NOP·C_3_H_8_O
*M* *_r_* = 353.38
Triclinic, 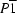

*a* = 9.0077 (9) Å
*b* = 11.7682 (12) Å
*c* = 18.7580 (18) Å
α = 78.717 (3)°
β = 79.169 (3)°
γ = 86.216 (2)°
*V* = 1914.4 (3) Å^3^
*Z* = 4
Mo *K*α radiation
μ = 0.16 mm^−1^
*T* = 120 (2) K
0.35 × 0.18 × 0.12 mm

#### Data collection

Bruker SMART 1000 CCD area-detector diffractometer
Absorption correction: none
18065 measured reflections
9090 independent reflections
6687 reflections with *I* > 2σ(*I*)
*R*_int_ = 0.022

#### Refinement

*R*[*F* ^2^ > 2σ(*F* ^2^)] = 0.061
*wR*(*F* ^2^) = 0.121
*S* = 1.03
9090 reflections
447 parameters
1 restraint
H-atom parameters constrained
Δρ_max_ = 1.04 e Å^−3^
Δρ_min_ = −0.71 e Å^−3^

### 

Data collection: *SMART* (Bruker, 2007 ▶); cell refinement: *SAINT-Plus* (Bruker, 2007 ▶); data reduction: *SAINT-Plus*; program(s) used to solve structure: *SHELXTL* (Sheldrick, 2008 ▶); program(s) used to refine structure: *SHELXTL*; molecular graphics: *SHELXTL*; software used to prepare material for publication: *SHELXTL*.

## Supplementary Material

Crystal structure: contains datablocks I, global. DOI: 10.1107/S1600536808004674/om2214sup1.cif

Structure factors: contains datablocks I. DOI: 10.1107/S1600536808004674/om2214Isup2.hkl

Additional supplementary materials:  crystallographic information; 3D view; checkCIF report

**Fig. 6.**
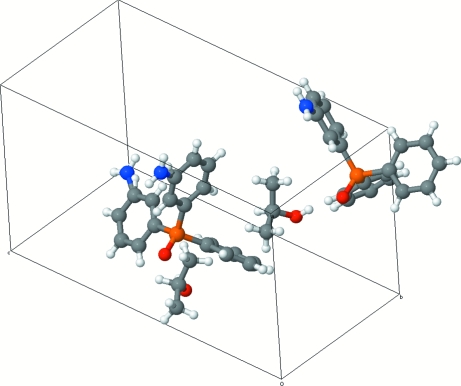
An interactive view of the asymmetric unit of the title compound including the disorder in the amino group position in the second molecule.

Enhanced figure: interactive version of Fig. 6

## Figures and Tables

**Table:** 

P1—O1	1.4974 (16)
P1—C7	1.799 (2)
P1—C13	1.802 (2)
P1—C1	1.803 (2)
P1′—O1′	1.4959 (16)
P1′—C1′	1.792 (2)
P1′—C7′	1.803 (2)
P1′—C13′	1.803 (2)

**Table:** 

O1—P1—C7	112.51 (10)
O1—P1—C13	112.56 (10)
C7—P1—C13	105.87 (10)
O1—P1—C1	112.47 (10)
C7—P1—C1	106.71 (10)
C13—P1—C1	106.21 (10)
O1′—P1′—C1′	112.49 (10)
O1′—P1′—C7′	111.81 (10)
C1′—P1′—C7′	106.37 (11)
O1′—P1′—C13′	110.02 (10)
C1′—P1′—C13′	108.52 (11)
C7′—P1′—C13′	107.41 (10)

**Table 2:** Hydrogen-bond geometry (Å, °)

*D*—H⋯*A*	*D*—H	H⋯*A*	*D*⋯*A*	*D*—H⋯*A*
N1—H1*A*⋯O1^i^	0.88	2.11	2.982 (3)	172
N1—H1*B*⋯O2*S*^ii^	0.88	2.19	3.051 (3)	165
O1*S*—H1*S*⋯O1′	0.84	1.84	2.680 (3)	179
O2*S*—H2*S*⋯O1	0.84	1.95	2.770 (3)	167
N1*A*—H1′*A*⋯O1′^iii^	0.88	2.16	2.970 (3)	154
N1*A*—H1′*B*⋯O1*S*^ii^	0.88	2.10	2.975 (3)	173
N1*B*—H1′*C*⋯O2*S*^iv^	0.88	2.10	2.869 (3)	146
C9—H9⋯O1*S*^v^	0.95	2.56	3.288 (3)	134
C14—H14⋯O2*S*	0.95	2.56	3.478 (3)	163
C3*S*—H3*SC*⋯*Cg*1^iii^	0.98	2.84	3.741 (4)	153
C4*S*—H4*SC*⋯*Cg*2^i^	0.98	2.93	3.645 (3)	131
N1*B*—H1′*D*⋯*Cg*3^iv^	0.98	2.45	3.296 (8)	161

Symmetry codes: (i) 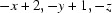; (ii) 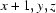; (iii) 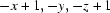; (iv) 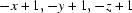; (v) 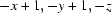. *Cg*1, *Cg*2 and *Cg*3 are the centroids of atoms C13′–C18′, C7–C12 and C7′–C12′, respectively.

**Fig. 1.**
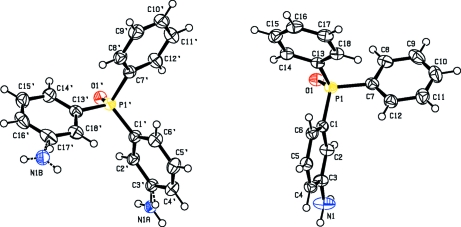
The molecular structure of the title compound, showing the atom-numbering scheme and displacements. Ellipsoids are drawn at 50% probability level. 2-propanol groups are removed for clarity. N1A and N1B atoms are disordered and occupancies of positions of atoms N1A and N1B are 0.75 and 1/4, respectively.

**Fig. 2.**
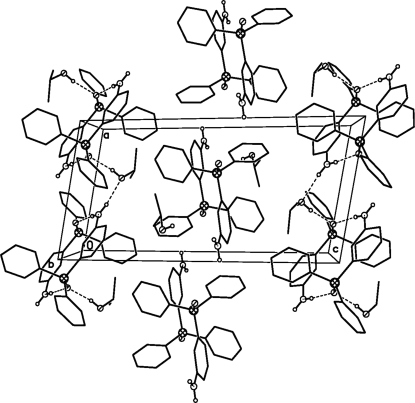
Crystal packing diagram of the title compound (along b crystal axis). Hydrogen atoms that do not take part in hydrogen bonding are not depicted for clarity. Hydrogen bonds are shown with dashed lines.

**Fig. 3.**
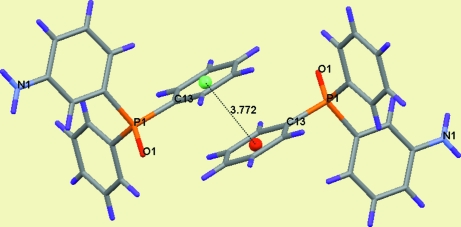
π-π Stacking interactions between aromatic rings of phenyl rings with distance of 3.7720 (15) Å [1 - x, 2 - y, -z].

**Fig. 4.**
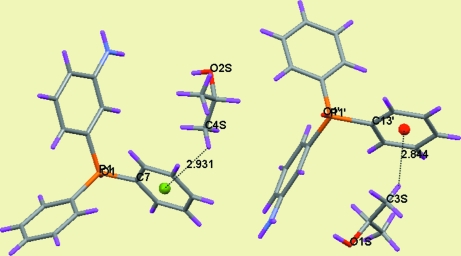
C—H···π Stacking interactions between C–H group of 2-propanol molecules with aromatic phenyl rings. The H···π distances (measured to the center of phenyl rings) is 2.84 Å for C3S—H3SC···Cg1 (1 - x, -y, 1 - z) and 2.93 Å for C4S—H4SC···Cg2 (2 - x, 1 - y, -z) [Cg1 and Cg2 are the centroids of C13'-C18' and C7—C12 rings, respectively].

**Fig. 5.**
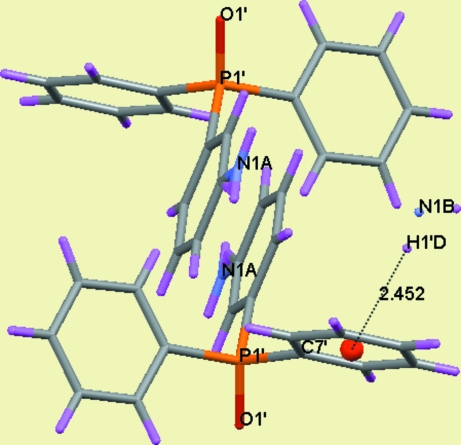
N—H···π Stacking interactions between between N–H group of aniline and phenyl rings with H···π distances of 2.45 Å for N1B—H1'D···Cg3 (1 - x, 1 - y, 1 - z) [Cg3 is the centroid for C7'-C12' ring].

## supplementary crystallographic information

### Comment

Polymer supported phosphine reagents have wide application in organic synthesis (Chael & Buchmeiser, 2003). Usually, for preparation of these reagents, mono functional derivatives of phosphine or phosphine oxide compounds are used. But direct mono-functionalization of triphenylphosphine and triphenylphosphine oxide is one of the problematic reactions in organic synthesis and generally higher functionalization is performed and di and/or tri- substituted products are obtained. In this study, the synthesis and characterization of 3-aminophenyldiphenylphosphine oxide is reported for the first time. The molecular structure of this compound is presented in Fig. 1, while the crystal packing diagram is illustrated in Fig. 2. Selected bond lengths and bond angles are presented in Table 1. Also hydrogen bond lengths are given separately in Table 2. This complex crystallizes in the triclinic system, space group *P*1, with four molecules in the unit cell. There are two symmetrically independent parts in the crystal structure. To each phosphorus atom are attached three C and one O atoms from two phenyl fragments, one aniline group and one double-bonded O atom. The phosphorus-oxygen bond distances are 1.4959 (16) and 1.4974 (16) Å, and phosphorus-carbon bond distances range from 1.792 (2) to 1.803 (2) Å, which are within normal ranges (Al-Farhan, 1992). According to the bond lengths and bond angles, arrangement of the four donor atoms around each phosphorus atom is distorted tetrahedral. N1A and N1B atoms are disordered, and occupancies of positions of atoms N1A and N1B are 0.75 and 1/4, respectively.

Significant π-π stacking interactions between aromatic rings of phenyl rings with a distance of 3.7720 (15) Å [1 - *x*, 2 - *y*, -*z*] is observed in the prepared compound (Fig. 3) (Aghabozorg, *et al.*, 2008).

Another noticeable feature of the title compound is the presence of C—H···π stacking interactions between C–H group of 2-propanol molecules with aromatic phenyl rings. The H···π distances (measured to the center of phenyl rings) is 2.84 Å for C3S—H3SC···*Cg*1 (1 - *x*, -*y*, 1 - *z*) and 2.93 Å for C4S—H4SC···*Cg*2 (2 - *x*, 1 - *y*, -*z*) [*Cg*1 and *Cg*2 are the centroids of C13'-C18' and C7—C12 rings, respectively] (Fig. 4) (Aghabozorg, *et al.*, 2007).

Also, a N—H···π stacking interaction between the N–H group of aniline and phenyl rings with H···π distances of 2.45 Å for N1B—H1'D···*Cg*3 (1 - *x*, 1 - *y*, 1 - *z*) [*Cg*3 is the centroid for C7'-C12' ring] is observed in the title compound (Fig. 5).

In the crystal structure of the title compound, a wide range of non-covalent interactions consisting of hydrogen bonding (of the types of O—H···O, N—H···O and C—H···O with D···A ranging from 2.680 (3) Å to 3.478 (3) Å), π-π [centroid–centroid distance of 3.7720 (15) Å], C—H···π and N—H···π stacking connect the various components into a supramolecular structure.

### Experimental

3-aminophenyldiphenylphosphine oxide (APDPPO) was prepared from triphenylphosphine oxide according to our previous paper (Mahdavi & Amani, 2008). In this procedure, the solid product was recrystallized from 2-propanol, and 3-aminophenyldiphenylphosphine oxide was obtained as white crystals. Yield was 87% with m.p. = 166 °C.

### Refinement

Hydrogen atoms on oxygen and nitrogen atoms were found from difference Fourier maps and on carbon atoms were placed in geometrically calculated positions. All hydrogen atoms were refined in isotropic approximation in riding model with the *U*_iso_(H) parameters equal to 1.5Ueq(O), 1.5Ueq(C) for methyl groups and to 1.2Ueq(N) and 1.2Ueq(C) for other carbon atoms where *U*_eq_(O), *U*_eq_(N) and *U*_eq_(C) are the equivalent thermal parameters of the atoms to which corresponding H atoms are bonded.

### Crystal data

**Table:**

C_18_H_16_NOP·C_3_H_8_O	*Z* = 4
*M**_r_* = 353.38	*F*_000_ = 752
Triclinic, *P*1	*D*_x_ = 1.226 Mg m^−^^3^
Hall symbol: -P 1	Mo *K*α radiation λ = 0.71073 Å
*a* = 9.0077 (9) Å	Cell parameters from 342 reflections
*b* = 11.7682 (12) Å	θ = 2.6–21.5º
*c* = 18.7580 (18) Å	µ = 0.16 mm^−^^1^
α = 78.717 (3)º	*T* = 120 (2) K
β = 79.169 (3)º	Prism, colourless
γ = 86.216 (2)º	0.35 × 0.18 × 0.12 mm
*V* = 1914.4 (3) Å^3^	

### Data collection

**Table:**

Bruker SMART 1000 CCD area-detector diffractometer	6687 reflections with *I* > 2σ(*I*)
Radiation source: fine-focus sealed tube	*R*_int_ = 0.022
Monochromator: graphite	θ_max_ = 28.0º
*T* = 120(2) K	θ_min_ = 1.8º
φ and ω scans	*h* = −11→11
Absorption correction: none	*k* = −15→15
18065 measured reflections	*l* = −24→24
9090 independent reflections	

### Refinement

**Table:**

Refinement on *F*^2^	Secondary atom site location: difference Fourier map
Least-squares matrix: full	Hydrogen site location: mixed
*R*[*F*^2^ > 2σ(*F*^2^)] = 0.061	H-atom parameters constrained
*wR*(*F*^2^) = 0.121	*w* = 1/[σ^2^(*F*_o_^2^) + (0.01*P*)^2^ + 2.8*P*] where *P* = (*F*_o_^2^ + 2*F*_c_^2^)/3
*S* = 1.03	(Δ/σ)_max_ < 0.001
9090 reflections	Δρ_max_ = 1.05 e Å^−^^3^
447 parameters	Δρ_min_ = −0.71 e Å^−^^3^
1 restraint	Extinction correction: none
Primary atom site location: structure-invariant direct methods	

### Special details

**Table:**

Geometry. All e.s.d.'s (except the e.s.d. in the dihedral angle between two l.s. planes) are estimated using the full covariance matrix. The cell e.s.d.'s are taken into account individually in the estimation of e.s.d.'s in distances, angles and torsion angles; correlations between e.s.d.'s in cell parameters are only used when they are defined by crystal symmetry. An approximate (isotropic) treatment of cell e.s.d.'s is used for estimating e.s.d.'s involving l.s. planes.
Refinement. Refinement of *F*^2^ against ALL reflections. The weighted *R*-factor *wR* and goodness of fit *S* are based on *F*^2^, conventional *R*-factors *R* are based on *F*, with *F* set to zero for negative *F*^2^. The threshold expression of *F*^2^ > σ(*F*^2^) is used only for calculating *R*-factors(gt) *etc*. and is not relevant to the choice of reflections for refinement. *R*-factors based on *F*^2^ are statistically about twice as large as those based on *F*, and *R*- factors based on ALL data will be even larger.

### Fractional atomic coordinates and isotropic or equivalent isotropic displacement parameters (Å^2^)

**Table:**

	*x*	*y*	*z*	*U*_iso_*/*U*_eq_	Occ. (<1)
P1	0.83874 (6)	0.74162 (5)	0.00875 (3)	0.02776 (13)	
O1	0.75714 (18)	0.63150 (13)	0.01769 (9)	0.0338 (4)	
C1	0.9933 (2)	0.72363 (19)	0.05909 (11)	0.0268 (4)	
C2	1.0938 (2)	0.62999 (19)	0.05107 (13)	0.0311 (5)	
H2	1.0794	0.5789	0.0197	0.037*	
C3	1.2161 (3)	0.6102 (2)	0.08876 (13)	0.0332 (5)	
C4	1.2350 (2)	0.6879 (2)	0.13397 (12)	0.0316 (5)	
H4	1.3176	0.6765	0.1598	0.038*	
C5	1.1353 (3)	0.7807 (2)	0.14148 (12)	0.0317 (5)	
H5	1.1505	0.8326	0.1722	0.038*	
C6	1.0130 (2)	0.7994 (2)	0.10472 (12)	0.0299 (5)	
H6A	0.9440	0.8629	0.1107	0.036*	
C7	0.9171 (3)	0.7982 (2)	−0.08617 (12)	0.0310 (5)	
C8	0.8423 (3)	0.7795 (2)	−0.14142 (14)	0.0387 (6)	
H8	0.7554	0.7332	−0.1291	0.046*	
C9	0.8958 (3)	0.8290 (3)	−0.21449 (14)	0.0490 (7)	
H9	0.8453	0.8160	−0.2522	0.059*	
C10	1.0219 (3)	0.8972 (2)	−0.23305 (14)	0.0472 (7)	
H10	1.0568	0.9314	−0.2832	0.057*	
C11	1.0970 (3)	0.9154 (2)	−0.17873 (14)	0.0433 (6)	
H11	1.1840	0.9615	−0.1913	0.052*	
C12	1.0443 (3)	0.8656 (2)	−0.10521 (13)	0.0357 (5)	
H12	1.0960	0.8780	−0.0678	0.043*	
C13	0.7173 (2)	0.85576 (19)	0.04114 (12)	0.0290 (5)	
C14	0.6066 (2)	0.8261 (2)	0.10429 (13)	0.0329 (5)	
H14	0.5939	0.7471	0.1274	0.039*	
C15	0.5157 (3)	0.9118 (2)	0.13308 (14)	0.0384 (6)	
H15	0.4412	0.8915	0.1762	0.046*	
C16	0.5328 (3)	1.0276 (2)	0.09912 (14)	0.0390 (6)	
H16	0.4704	1.0861	0.1192	0.047*	
C17	0.6404 (3)	1.0575 (2)	0.03624 (14)	0.0368 (5)	
H17	0.6512	1.1364	0.0127	0.044*	
C18	0.7329 (3)	0.9719 (2)	0.00737 (13)	0.0322 (5)	
H18	0.8073	0.9928	−0.0357	0.039*	
N1	1.3129 (3)	0.5171 (2)	0.08285 (14)	0.0525 (6)	
H1A	1.2991	0.4680	0.0550	0.063*	
H1B	1.3891	0.5060	0.1069	0.063*	
P1'	0.37636 (6)	0.24882 (5)	0.48023 (3)	0.02777 (13)	
O1'	0.31222 (17)	0.13607 (13)	0.47740 (8)	0.0318 (3)	
C1'	0.5759 (2)	0.2540 (2)	0.44592 (12)	0.0307 (2)	
C2'	0.6575 (2)	0.1492 (2)	0.45007 (12)	0.0307 (2)	
H2'	0.6065	0.0786	0.4691	0.037*	
C3'	0.8148 (2)	0.1471 (2)	0.42637 (12)	0.0307 (2)	
H3'	0.9012	0.0947	0.4246	0.037*	0.25
C4'	0.8873 (3)	0.2528 (2)	0.39855 (14)	0.0381 (5)	
H4'	0.9939	0.2531	0.3829	0.046*	
C5'	0.8047 (3)	0.3564 (2)	0.39383 (14)	0.0407 (6)	
H5'	0.8551	0.4272	0.3742	0.049*	
C6'	0.6485 (3)	0.3584 (2)	0.41752 (14)	0.0374 (5)	
H6'	0.5924	0.4299	0.4144	0.045*	
C7'	0.2912 (2)	0.36956 (19)	0.42528 (13)	0.0310 (5)	
C8'	0.2197 (3)	0.4637 (2)	0.45372 (14)	0.0374 (5)	
H8'	0.2167	0.4674	0.5041	0.045*	
C9'	0.1527 (3)	0.5523 (2)	0.40871 (16)	0.0468 (7)	
H9'	0.1043	0.6166	0.4283	0.056*	
C10'	0.1562 (3)	0.5472 (2)	0.33547 (16)	0.0492 (7)	
H10'	0.1100	0.6079	0.3050	0.059*	
C11'	0.2270 (3)	0.4538 (2)	0.30614 (15)	0.0486 (7)	
H11'	0.2297	0.4507	0.2557	0.058*	
C12'	0.2938 (3)	0.3649 (2)	0.35120 (14)	0.0406 (6)	
H12'	0.3416	0.3006	0.3315	0.049*	
C13'	0.3417 (3)	0.27312 (19)	0.57398 (12)	0.0305 (5)	
C14'	0.2074 (3)	0.2318 (2)	0.61931 (14)	0.0404 (6)	
H14'	0.1383	0.1936	0.6001	0.048*	
C15'	0.1748 (3)	0.2464 (2)	0.69210 (14)	0.0474 (7)	
H15'	0.0835	0.2178	0.7227	0.057*	
C16'	0.2733 (3)	0.3018 (2)	0.72049 (14)	0.0486 (7)	
H16'	0.2494	0.3126	0.7703	0.058*	
C17'	0.4081 (3)	0.3420 (2)	0.67617 (15)	0.0449 (6)	
H17'	0.4715	0.3904	0.6925	0.054*	0.75
C18'	0.4429 (3)	0.3276 (2)	0.60274 (14)	0.0367 (5)	
H18'	0.5353	0.3548	0.5726	0.044*	
O1S	0.2229 (2)	0.05513 (18)	0.36881 (11)	0.0506 (5)	
H1S	0.2515	0.0802	0.4028	0.076*	
O2S	0.5924 (2)	0.52560 (15)	0.15145 (11)	0.0453 (4)	
H2S	0.6522	0.5489	0.1119	0.068*	
C1S	0.2430 (3)	−0.1203 (3)	0.3254 (2)	0.0687 (10)	
H1SA	0.1520	−0.1437	0.3618	0.103*	
H1SB	0.3090	−0.1886	0.3191	0.103*	
H1SC	0.2141	−0.0846	0.2780	0.103*	
C2S	0.3203 (3)	−0.0401 (3)	0.3501 (2)	0.0767 (12)	
H2SA	0.3334	−0.0835	0.4001	0.092*	
C3S	0.4742 (3)	−0.0061 (3)	0.31770 (17)	0.0527 (7)	
H3SA	0.5162	0.0297	0.3522	0.079*	
H3SB	0.4739	0.0496	0.2715	0.079*	
H3SC	0.5361	−0.0747	0.3076	0.079*	
C4S	0.8208 (3)	0.4225 (2)	0.18314 (17)	0.0494 (7)	
H4SA	0.8470	0.4880	0.2031	0.074*	
H4SB	0.8686	0.4307	0.1310	0.074*	
H4SC	0.8570	0.3500	0.2110	0.074*	
C5S	0.6520 (4)	0.4207 (3)	0.1897 (2)	0.0605 (8)	
H5S	0.6096	0.4179	0.2433	0.073*	
C6S	0.5949 (4)	0.3199 (3)	0.1707 (3)	0.0936 (15)	
H6SA	0.4842	0.3255	0.1783	0.140*	
H6SB	0.6269	0.2494	0.2022	0.140*	
H6SC	0.6351	0.3168	0.1188	0.140*	
N1A	0.8929 (3)	0.0434 (2)	0.42844 (13)	0.0307 (2)	0.75
H1'A	0.8445	−0.0218	0.4446	0.037*	0.75
H1'B	0.9915	0.0418	0.4136	0.037*	0.75
N1B	0.4942 (9)	0.3810 (8)	0.7160 (4)	0.044 (2)	0.25
H1'C	0.4622	0.3787	0.7635	0.052*	0.25
H1'D	0.5832	0.4091	0.6947	0.052*	0.25

### Atomic displacement parameters (Å^2^)

**Table:**

	*U*^11^	*U*^22^	*U*^33^	*U*^12^	*U*^13^	*U*^23^
P1	0.0276 (3)	0.0284 (3)	0.0296 (3)	0.0010 (2)	−0.0083 (2)	−0.0085 (2)
O1	0.0331 (8)	0.0300 (8)	0.0415 (9)	−0.0020 (7)	−0.0116 (7)	−0.0097 (7)
C1	0.0269 (10)	0.0272 (11)	0.0258 (10)	−0.0008 (8)	−0.0060 (8)	−0.0030 (8)
C2	0.0322 (11)	0.0272 (11)	0.0374 (12)	0.0011 (9)	−0.0114 (9)	−0.0100 (9)
C3	0.0311 (11)	0.0279 (12)	0.0422 (13)	0.0018 (9)	−0.0120 (10)	−0.0062 (10)
C4	0.0292 (11)	0.0354 (12)	0.0315 (11)	−0.0037 (9)	−0.0105 (9)	−0.0041 (9)
C5	0.0341 (12)	0.0340 (12)	0.0293 (11)	−0.0041 (10)	−0.0065 (9)	−0.0097 (9)
C6	0.0302 (11)	0.0311 (12)	0.0291 (11)	0.0000 (9)	−0.0041 (9)	−0.0084 (9)
C7	0.0328 (11)	0.0310 (12)	0.0309 (11)	0.0079 (9)	−0.0086 (9)	−0.0099 (9)
C8	0.0401 (13)	0.0415 (14)	0.0396 (13)	0.0093 (11)	−0.0164 (11)	−0.0142 (11)
C9	0.0644 (18)	0.0542 (17)	0.0334 (13)	0.0206 (14)	−0.0211 (13)	−0.0157 (12)
C10	0.0640 (18)	0.0429 (15)	0.0291 (12)	0.0165 (13)	−0.0030 (12)	−0.0046 (11)
C11	0.0476 (15)	0.0393 (14)	0.0380 (14)	0.0065 (12)	0.0003 (11)	−0.0055 (11)
C12	0.0382 (13)	0.0373 (13)	0.0325 (12)	0.0037 (10)	−0.0078 (10)	−0.0087 (10)
C13	0.0273 (10)	0.0296 (12)	0.0328 (11)	0.0022 (9)	−0.0114 (9)	−0.0079 (9)
C14	0.0309 (11)	0.0337 (12)	0.0353 (12)	−0.0005 (9)	−0.0086 (9)	−0.0072 (10)
C15	0.0321 (12)	0.0447 (15)	0.0390 (13)	0.0023 (10)	−0.0031 (10)	−0.0136 (11)
C16	0.0345 (12)	0.0390 (14)	0.0502 (15)	0.0085 (10)	−0.0129 (11)	−0.0222 (12)
C17	0.0375 (13)	0.0304 (12)	0.0465 (14)	0.0040 (10)	−0.0160 (11)	−0.0104 (11)
C18	0.0300 (11)	0.0305 (12)	0.0374 (12)	0.0010 (9)	−0.0092 (9)	−0.0068 (10)
N1	0.0472 (13)	0.0432 (13)	0.0825 (18)	0.0155 (10)	−0.0363 (13)	−0.0306 (13)
P1'	0.0287 (3)	0.0265 (3)	0.0286 (3)	0.0001 (2)	−0.0047 (2)	−0.0071 (2)
O1'	0.0349 (8)	0.0286 (8)	0.0329 (8)	−0.0038 (7)	−0.0064 (7)	−0.0067 (7)
C1'	0.0288 (6)	0.0307 (6)	0.0318 (6)	0.0006 (5)	−0.0029 (5)	−0.0064 (5)
C2'	0.0288 (6)	0.0307 (6)	0.0318 (6)	0.0006 (5)	−0.0029 (5)	−0.0064 (5)
C3'	0.0288 (6)	0.0307 (6)	0.0318 (6)	0.0006 (5)	−0.0029 (5)	−0.0064 (5)
C4'	0.0298 (12)	0.0423 (14)	0.0415 (13)	−0.0042 (10)	0.0001 (10)	−0.0116 (11)
C5'	0.0399 (13)	0.0350 (13)	0.0456 (14)	−0.0105 (11)	0.0013 (11)	−0.0090 (11)
C6'	0.0379 (13)	0.0288 (12)	0.0443 (14)	−0.0012 (10)	−0.0030 (11)	−0.0080 (10)
C7'	0.0291 (11)	0.0274 (11)	0.0350 (12)	−0.0026 (9)	−0.0048 (9)	−0.0029 (9)
C8'	0.0348 (12)	0.0351 (13)	0.0395 (13)	0.0018 (10)	−0.0010 (10)	−0.0065 (10)
C9'	0.0410 (14)	0.0365 (14)	0.0558 (17)	0.0088 (11)	−0.0005 (12)	−0.0023 (12)
C10'	0.0429 (15)	0.0443 (16)	0.0531 (17)	0.0045 (12)	−0.0103 (12)	0.0082 (13)
C11'	0.0579 (17)	0.0479 (16)	0.0384 (14)	−0.0005 (13)	−0.0147 (12)	0.0015 (12)
C12'	0.0488 (15)	0.0361 (14)	0.0373 (13)	0.0011 (11)	−0.0111 (11)	−0.0054 (11)
C13'	0.0349 (12)	0.0289 (12)	0.0293 (11)	0.0061 (9)	−0.0090 (9)	−0.0081 (9)
C14'	0.0374 (13)	0.0453 (15)	0.0367 (13)	0.0040 (11)	−0.0034 (10)	−0.0087 (11)
C15'	0.0502 (16)	0.0523 (17)	0.0340 (13)	0.0109 (13)	0.0009 (12)	−0.0068 (12)
C16'	0.0722 (19)	0.0440 (15)	0.0290 (12)	0.0232 (14)	−0.0110 (13)	−0.0116 (11)
C17'	0.0669 (18)	0.0321 (13)	0.0432 (14)	0.0129 (12)	−0.0266 (13)	−0.0144 (11)
C18'	0.0430 (13)	0.0297 (12)	0.0397 (13)	0.0035 (10)	−0.0131 (11)	−0.0082 (10)
O1S	0.0404 (10)	0.0664 (13)	0.0573 (12)	0.0169 (9)	−0.0174 (9)	−0.0394 (10)
O2S	0.0388 (10)	0.0368 (10)	0.0562 (11)	0.0010 (8)	−0.0118 (8)	0.0042 (8)
C1S	0.0431 (16)	0.0442 (17)	0.119 (3)	−0.0012 (13)	−0.0005 (18)	−0.0284 (19)
C2S	0.0361 (15)	0.078 (2)	0.133 (3)	0.0082 (15)	−0.0062 (18)	−0.074 (2)
C3S	0.0418 (15)	0.0530 (17)	0.0663 (19)	−0.0015 (13)	−0.0002 (13)	−0.0269 (15)
C4S	0.0445 (15)	0.0444 (16)	0.0588 (18)	0.0089 (12)	−0.0130 (13)	−0.0085 (13)
C5S	0.0520 (17)	0.0431 (17)	0.084 (2)	−0.0022 (14)	−0.0248 (16)	0.0059 (16)
C6S	0.059 (2)	0.0368 (18)	0.188 (5)	0.0037 (16)	−0.039 (3)	−0.014 (2)
N1A	0.0288 (6)	0.0307 (6)	0.0318 (6)	0.0006 (5)	−0.0029 (5)	−0.0064 (5)
N1B	0.039 (5)	0.056 (6)	0.037 (5)	−0.011 (4)	−0.008 (4)	−0.006 (4)

### Geometric parameters (Å, °)

**Table:**

P1—O1	1.4974 (16)	C5'—C6'	1.393 (3)
P1—C7	1.799 (2)	C5'—H5'	0.9500
P1—C13	1.802 (2)	C6'—H6'	0.9500
P1—C1	1.803 (2)	C7'—C8'	1.391 (3)
C1—C6	1.391 (3)	C7'—C12'	1.397 (3)
C1—C2	1.393 (3)	C8'—C9'	1.389 (4)
C2—C3	1.400 (3)	C8'—H8'	0.9500
C2—H2	0.9500	C9'—C10'	1.381 (4)
C3—N1	1.364 (3)	C9'—H9'	0.9500
C3—C4	1.401 (3)	C10'—C11'	1.389 (4)
C4—C5	1.379 (3)	C10'—H10'	0.9500
C4—H4	0.9500	C11'—C12'	1.390 (4)
C5—C6	1.390 (3)	C11'—H11'	0.9500
C5—H5	0.9500	C12'—H12'	0.9500
C6—H6A	0.9500	C13'—C18'	1.389 (3)
C7—C12	1.387 (3)	C13'—C14'	1.397 (3)
C7—C8	1.395 (3)	C14'—C15'	1.384 (3)
C8—C9	1.388 (4)	C14'—H14'	0.9500
C8—H8	0.9500	C15'—C16'	1.373 (4)
C9—C10	1.385 (4)	C15'—H15'	0.9500
C9—H9	0.9500	C16'—C17'	1.390 (4)
C10—C11	1.380 (4)	C16'—H16'	0.9500
C10—H10	0.9500	C17'—N1B	1.329 (4)
C11—C12	1.394 (3)	C17'—C18'	1.395 (3)
C11—H11	0.9500	C17'—H17'	0.9601
C12—H12	0.9500	C18'—H18'	0.9500
C13—C18	1.393 (3)	O1S—C2S	1.434 (3)
C13—C14	1.400 (3)	O1S—H1S	0.8400
C14—C15	1.385 (3)	O2S—C5S	1.428 (3)
C14—H14	0.9500	O2S—H2S	0.8400
C15—C16	1.392 (4)	C1S—C2S	1.403 (4)
C15—H15	0.9500	C1S—H1SA	0.9800
C16—C17	1.381 (4)	C1S—H1SB	0.9800
C16—H16	0.9500	C1S—H1SC	0.9800
C17—C18	1.391 (3)	C2S—C3S	1.454 (4)
C17—H17	0.9500	C2S—H2SA	1.0000
C18—H18	0.9500	C3S—H3SA	0.9800
N1—H1A	0.8800	C3S—H3SB	0.9800
N1—H1B	0.8800	C3S—H3SC	0.9800
P1'—O1'	1.4959 (16)	C4S—C5S	1.504 (4)
P1'—C1'	1.792 (2)	C4S—H4SA	0.9800
P1'—C7'	1.803 (2)	C4S—H4SB	0.9800
P1'—C13'	1.803 (2)	C4S—H4SC	0.9800
C1'—C2'	1.389 (3)	C5S—C6S	1.453 (5)
C1'—C6'	1.394 (3)	C5S—H5S	1.0000
C2'—C3'	1.403 (3)	C6S—H6SA	0.9800
C2'—H2'	0.9500	C6S—H6SB	0.9800
C3'—N1A	1.364 (3)	C6S—H6SC	0.9800
C3'—C4'	1.404 (3)	N1A—H1'A	0.8800
C3'—H3'	0.9600	N1A—H1'B	0.8800
C4'—C5'	1.383 (4)	N1B—H1'C	0.8800
C4'—H4'	0.9500	N1B—H1'D	0.8800
			
O1—P1—C7	112.51 (10)	C5'—C6'—C1'	119.0 (2)
O1—P1—C13	112.56 (10)	C5'—C6'—H6'	120.5
C7—P1—C13	105.87 (10)	C1'—C6'—H6'	120.5
O1—P1—C1	112.47 (10)	C8'—C7'—C12'	119.2 (2)
C7—P1—C1	106.71 (10)	C8'—C7'—P1'	123.25 (18)
C13—P1—C1	106.21 (10)	C12'—C7'—P1'	117.48 (18)
C6—C1—C2	120.4 (2)	C9'—C8'—C7'	120.2 (2)
C6—C1—P1	122.05 (17)	C9'—C8'—H8'	119.9
C2—C1—P1	117.58 (16)	C7'—C8'—H8'	119.9
C1—C2—C3	120.8 (2)	C10'—C9'—C8'	120.1 (3)
C1—C2—H2	119.6	C10'—C9'—H9'	119.9
C3—C2—H2	119.6	C8'—C9'—H9'	119.9
N1—C3—C2	121.2 (2)	C9'—C10'—C11'	120.5 (3)
N1—C3—C4	120.6 (2)	C9'—C10'—H10'	119.8
C2—C3—C4	118.1 (2)	C11'—C10'—H10'	119.8
C5—C4—C3	120.8 (2)	C10'—C11'—C12'	119.5 (3)
C5—C4—H4	119.6	C10'—C11'—H11'	120.3
C3—C4—H4	119.6	C12'—C11'—H11'	120.3
C4—C5—C6	120.9 (2)	C11'—C12'—C7'	120.5 (2)
C4—C5—H5	119.5	C11'—C12'—H12'	119.8
C6—C5—H5	119.5	C7'—C12'—H12'	119.8
C5—C6—C1	119.0 (2)	C18'—C13'—C14'	119.6 (2)
C5—C6—H6A	120.5	C18'—C13'—P1'	123.20 (18)
C1—C6—H6A	120.5	C14'—C13'—P1'	117.21 (18)
C12—C7—C8	119.4 (2)	C15'—C14'—C13'	120.1 (3)
C12—C7—P1	121.55 (17)	C15'—C14'—H14'	119.9
C8—C7—P1	118.90 (19)	C13'—C14'—H14'	119.9
C9—C8—C7	119.6 (3)	C16'—C15'—C14'	120.6 (3)
C9—C8—H8	120.2	C16'—C15'—H15'	119.7
C7—C8—H8	120.2	C14'—C15'—H15'	119.7
C10—C9—C8	120.7 (2)	C15'—C16'—C17'	119.8 (2)
C10—C9—H9	119.6	C15'—C16'—H16'	120.1
C8—C9—H9	119.6	C17'—C16'—H16'	120.1
C11—C10—C9	120.0 (2)	N1B—C17'—C16'	110.4 (4)
C11—C10—H10	120.0	N1B—C17'—C18'	129.0 (5)
C9—C10—H10	120.0	C16'—C17'—C18'	120.3 (2)
C10—C11—C12	119.6 (3)	C16'—C17'—H17'	121.0
C10—C11—H11	120.2	C18'—C17'—H17'	117.9
C12—C11—H11	120.2	C13'—C18'—C17'	119.6 (2)
C7—C12—C11	120.6 (2)	C13'—C18'—H18'	120.2
C7—C12—H12	119.7	C17'—C18'—H18'	120.2
C11—C12—H12	119.7	C2S—O1S—H1S	109.5
C18—C13—C14	119.2 (2)	C5S—O2S—H2S	109.5
C18—C13—P1	122.61 (17)	C2S—C1S—H1SA	109.5
C14—C13—P1	118.16 (17)	C2S—C1S—H1SB	109.5
C15—C14—C13	120.0 (2)	H1SA—C1S—H1SB	109.5
C15—C14—H14	120.0	C2S—C1S—H1SC	109.5
C13—C14—H14	120.0	H1SA—C1S—H1SC	109.5
C14—C15—C16	120.3 (2)	H1SB—C1S—H1SC	109.5
C14—C15—H15	119.9	C1S—C2S—O1S	111.7 (3)
C16—C15—H15	119.9	C1S—C2S—C3S	123.7 (3)
C17—C16—C15	120.0 (2)	O1S—C2S—C3S	113.0 (3)
C17—C16—H16	120.0	C1S—C2S—H2SA	101.5
C15—C16—H16	120.0	O1S—C2S—H2SA	101.5
C16—C17—C18	119.9 (2)	C3S—C2S—H2SA	101.5
C16—C17—H17	120.0	C2S—C3S—H3SA	109.5
C18—C17—H17	120.0	C2S—C3S—H3SB	109.5
C17—C18—C13	120.5 (2)	H3SA—C3S—H3SB	109.5
C17—C18—H18	119.8	C2S—C3S—H3SC	109.5
C13—C18—H18	119.8	H3SA—C3S—H3SC	109.5
C3—N1—H1A	120.0	H3SB—C3S—H3SC	109.5
C3—N1—H1B	120.0	C5S—C4S—H4SA	109.5
H1A—N1—H1B	120.0	C5S—C4S—H4SB	109.5
O1'—P1'—C1'	112.49 (10)	H4SA—C4S—H4SB	109.5
O1'—P1'—C7'	111.81 (10)	C5S—C4S—H4SC	109.5
C1'—P1'—C7'	106.37 (11)	H4SA—C4S—H4SC	109.5
O1'—P1'—C13'	110.02 (10)	H4SB—C4S—H4SC	109.5
C1'—P1'—C13'	108.52 (11)	O2S—C5S—C6S	111.1 (3)
C7'—P1'—C13'	107.41 (10)	O2S—C5S—C4S	112.0 (2)
C2'—C1'—C6'	120.6 (2)	C6S—C5S—C4S	115.7 (3)
C2'—C1'—P1'	117.44 (17)	O2S—C5S—H5S	105.8
C6'—C1'—P1'	121.93 (18)	C6S—C5S—H5S	105.8
C1'—C2'—C3'	120.5 (2)	C4S—C5S—H5S	105.8
C1'—C2'—H2'	119.8	C5S—C6S—H6SA	109.5
C3'—C2'—H2'	119.8	C5S—C6S—H6SB	109.5
N1A—C3'—C2'	119.8 (2)	H6SA—C6S—H6SB	109.5
N1A—C3'—C4'	121.6 (2)	C5S—C6S—H6SC	109.5
C2'—C3'—C4'	118.6 (2)	H6SA—C6S—H6SC	109.5
C2'—C3'—H3'	141.5	H6SB—C6S—H6SC	109.5
C4'—C3'—H3'	99.8	C3'—N1A—H1'A	120.0
C5'—C4'—C3'	120.5 (2)	C3'—N1A—H1'B	120.0
C5'—C4'—H4'	119.8	H1'A—N1A—H1'B	120.0
C3'—C4'—H4'	119.8	C17'—N1B—H1'C	120.0
C4'—C5'—C6'	120.9 (2)	C17'—N1B—H1'D	120.0
C4'—C5'—H5'	119.6	H1'C—N1B—H1'D	120.0
C6'—C5'—H5'	119.6		
			
O1—P1—C1—C6	−131.38 (18)	C7'—P1'—C1'—C2'	−148.70 (18)
C7—P1—C1—C6	104.79 (19)	C13'—P1'—C1'—C2'	96.01 (19)
C13—P1—C1—C6	−7.8 (2)	O1'—P1'—C1'—C6'	155.42 (19)
O1—P1—C1—C2	48.4 (2)	C7'—P1'—C1'—C6'	32.7 (2)
C7—P1—C1—C2	−75.40 (19)	C13'—P1'—C1'—C6'	−82.6 (2)
C13—P1—C1—C2	171.98 (17)	C6'—C1'—C2'—C3'	0.6 (3)
C6—C1—C2—C3	0.1 (3)	P1'—C1'—C2'—C3'	−177.99 (17)
P1—C1—C2—C3	−179.68 (18)	C1'—C2'—C3'—N1A	−177.8 (2)
C1—C2—C3—N1	178.2 (2)	C1'—C2'—C3'—C4'	0.0 (3)
C1—C2—C3—C4	−0.7 (3)	N1A—C3'—C4'—C5'	176.9 (2)
N1—C3—C4—C5	−178.5 (2)	C2'—C3'—C4'—C5'	−0.8 (4)
C2—C3—C4—C5	0.4 (3)	C3'—C4'—C5'—C6'	1.0 (4)
C3—C4—C5—C6	0.3 (3)	C4'—C5'—C6'—C1'	−0.3 (4)
C4—C5—C6—C1	−0.9 (3)	C2'—C1'—C6'—C5'	−0.5 (4)
C2—C1—C6—C5	0.6 (3)	P1'—C1'—C6'—C5'	178.07 (19)
P1—C1—C6—C5	−179.56 (17)	O1'—P1'—C7'—C8'	123.4 (2)
O1—P1—C7—C12	−152.32 (18)	C1'—P1'—C7'—C8'	−113.4 (2)
C13—P1—C7—C12	84.3 (2)	C13'—P1'—C7'—C8'	2.6 (2)
C1—P1—C7—C12	−28.5 (2)	O1'—P1'—C7'—C12'	−54.4 (2)
O1—P1—C7—C8	31.6 (2)	C1'—P1'—C7'—C12'	68.8 (2)
C13—P1—C7—C8	−91.7 (2)	C13'—P1'—C7'—C12'	−175.16 (19)
C1—P1—C7—C8	155.44 (18)	C12'—C7'—C8'—C9'	−0.4 (4)
C12—C7—C8—C9	−0.3 (3)	P1'—C7'—C8'—C9'	−178.18 (19)
P1—C7—C8—C9	175.80 (19)	C7'—C8'—C9'—C10'	0.2 (4)
C7—C8—C9—C10	−0.3 (4)	C8'—C9'—C10'—C11'	−0.2 (4)
C8—C9—C10—C11	0.8 (4)	C9'—C10'—C11'—C12'	0.3 (4)
C9—C10—C11—C12	−0.5 (4)	C10'—C11'—C12'—C7'	−0.5 (4)
C8—C7—C12—C11	0.6 (3)	C8'—C7'—C12'—C11'	0.6 (4)
P1—C7—C12—C11	−175.48 (18)	P1'—C7'—C12'—C11'	178.5 (2)
C10—C11—C12—C7	−0.1 (4)	O1'—P1'—C13'—C18'	144.78 (19)
O1—P1—C13—C18	−145.04 (18)	C1'—P1'—C13'—C18'	21.3 (2)
C7—P1—C13—C18	−21.7 (2)	C7'—P1'—C13'—C18'	−93.3 (2)
C1—P1—C13—C18	91.5 (2)	O1'—P1'—C13'—C14'	−33.6 (2)
O1—P1—C13—C14	37.2 (2)	C1'—P1'—C13'—C14'	−157.07 (18)
C7—P1—C13—C14	160.52 (17)	C7'—P1'—C13'—C14'	88.3 (2)
C1—P1—C13—C14	−86.28 (19)	C18'—C13'—C14'—C15'	0.8 (4)
C18—C13—C14—C15	−0.9 (3)	P1'—C13'—C14'—C15'	179.2 (2)
P1—C13—C14—C15	176.95 (18)	C13'—C14'—C15'—C16'	0.3 (4)
C13—C14—C15—C16	0.6 (4)	C14'—C15'—C16'—C17'	−1.1 (4)
C14—C15—C16—C17	0.3 (4)	C15'—C16'—C17'—N1B	−173.1 (5)
C15—C16—C17—C18	−0.8 (4)	C15'—C16'—C17'—C18'	0.8 (4)
C16—C17—C18—C13	0.4 (3)	C14'—C13'—C18'—C17'	−1.0 (3)
C14—C13—C18—C17	0.4 (3)	P1'—C13'—C18'—C17'	−179.36 (18)
P1—C13—C18—C17	−177.34 (17)	N1B—C17'—C18'—C13'	172.9 (6)
O1'—P1'—C1'—C2'	−26.0 (2)	C16'—C17'—C18'—C13'	0.2 (4)

### Hydrogen-bond geometry (Å, °)

**Table:**

*D*—H···*A*	*D*—H	H···*A*	*D*···*A*	*D*—H···*A*
N1—H1A···O1^i^	0.88	2.11	2.982 (3)	172
N1—H1B···O2S^ii^	0.88	2.19	3.051 (3)	165
O1S—H1S···O1'	0.84	1.84	2.680 (3)	179
O2S—H2S···O1	0.84	1.95	2.770 (3)	167
N1A—H1'A···O1'^iii^	0.88	2.16	2.970 (3)	154
N1A—H1'B···O1S^ii^	0.88	2.10	2.975 (3)	173
N1B—H1'C···O2S^iv^	0.88	2.10	2.869 (3)	146
C9—H9···O1S^v^	0.95	2.56	3.288 (3)	134
C14—H14···O2S	0.95	2.56	3.478 (3)	163
C3S—H3SC···Cg1^iii^	0.98	2.84	3.741 (4)	153
C4S—H4SC···Cg2^i^	0.98	2.93	3.645 (3)	131
N1B—H1'D···Cg3^iv^	0.88	2.45	3.296 (8)	161

Symmetry codes: (i) −*x*+2, −*y*+1, −*z*; (ii) *x*+1, *y*, *z*;  (iii) −*x*+1, −*y*, −*z*+1; (iv) −*x*+1, −*y*+1, −*z*+1; (v) −*x*+1, −*y*+1, −*z*.
